# Genome-Wide Identification of the *BPC* Gene Family in *Brassica juncea* and Expression Analysis of Its Regulatory Mechanisms in Response to Light and Salicylic Acid

**DOI:** 10.3390/ijms27062664

**Published:** 2026-03-14

**Authors:** Shunlin Wang, Zewen Lu, Jiahui Bai, Yujia Chen, Yang Yang, Guoping Shu, Changgui Yang, Zengxiang Wu, Pengfei Li

**Affiliations:** 1 Institute of Chinese Materia Medica and Ethnomedicine Resources, Provincial Key Laboratory of Germplasm Innovation and Efficient Utilization of Authentic Medicinal Materials, Guizhou University of Traditional Chinese Medicine, Guiyang 550025, China; wangshunlin0353@163.com (S.W.);; 2 Guangdong Key Laboratory for New Technology Research of Vegetables, Vegetable Research Institute, Guangdong Academy of Agricultural Sciences, Guangzhou 510640, China

**Keywords:** *Brassica juncea*, *BPC*, gene family, genome-wide identification, expression pattern

## Abstract

BASIC PENTACYSTEINE (*BPC*) transcription factors are plant-specific and play crucial roles in regulating plant development and responses to abiotic stresses. However, the genomic characteristics of the *BPC* gene family in *Brassica juncea* and its regulatory mechanisms in response to light and salicylic acid remain poorly understood. In this study, we identified 25 *BjuBPC* genes in the *B. juncea* genome using bioinformatic approaches. All BjuBPC proteins were predicted to localize exclusively to the nucleus, with their distribution scattered across 14 chromosomes of *B. juncea*. Phylogenetic analysis classified these *BjuBPC* genes into three subfamilies (A, B, and C). The 25 *BjuBPC* genes showed strong collinearity with *BPC* orthologs from *Arabidopsis thaliana*, *Brassica rapa*, and *Brassica nigra*, and members of the same subfamily shared highly conserved exon–intron architectures and motif compositions, and a highly conserved canonical GAGA DNA-binding domain. Expression profiling across tissues revealed both tissue-specific and constitutive expression patterns among *BjuBPC* members. Subsequent expression analyses under four light qualities and exogenous salicylic acid treatment demonstrated that *BjuBPC1*, *BjuBPC9*, and *BjuBPC24* were specifically responsive to both light and salicylic acid signals, with markedly strong induction by blue light. These findings provide valuable insights for future functional characterization of *BjuBPC* genes and enhance our understanding of their biological roles in *B. juncea*.

## 1. Introduction

Polyploidization is a key driving force underlying plant speciation and evolutionary processes [1,2,3]. Compared with diploid species, polyploids often exhibit enhanced yield potential, greater environmental adaptability, and richer genetic diversity [4,5]. These polyploids not only provide unique genetic resources for studying chromosomal structural variation, subgenome interactions, and gene function, but also lay a foundation for crop molecular breeding. *Brassica juncea* (AABB, 2*n* = 36), an allotetraploid species within the genus *Brassica*, originated from natural hybridization between the diploid progenitors *B. rapa* (AA, 2*n* = 20) and *B. nigra* (BB, 2*n* = 16) approximately 8000–14,000 years ago in West Asia [6]. Through subsequent mutations and introgressive hybridization, *B. juncea* has diversified into six distinct genetic groups, making it a globally important dual-purpose crop for oil and vegetable production [6,7]. Moreover, its seeds are listed in the *Pharmacopoeia of the People’s Republic of China* (2025 edition) as the botanical source of the traditional Chinese medicine “Jie Zi”, which is used to warm the lungs, resolve phlegm, promote “Qi” circulation, dissipate nodules, and relieve pain.

Compared with hexaploid wheat or other cereal crops, *B. juncea* has a relatively smaller genome and a clearer genetic background, making it more suitable for the precise identification, localization, and evolutionary analysis of gene families [8,9]. Meanwhile, as a typical polyploid crop, *B. juncea* is widely cultivated in arid, semi-arid, and saline-alkali regions, exhibiting strong tolerance to various abiotic stresses, and thus represents an ideal material for studying stress adaptation mechanisms in polyploid crops [10,11]. In modern agriculture, identifying key transcription factors—such as *MYB*, *BPC*, *DOF* and *AP2*/*ERF*—that regulate growth, development, and stress responses and leveraging this molecular knowledge to develop crops with improved stress resilience (e.g., lodging resistance, disease/pest tolerance, salinity/alkalinity tolerance) and ideal plant architecture (e.g., high-density suitability, mechanized harvest compatibility, and high biomass) [12,13,14,15] have become a major focus [16,17,18,19,20]. Among these regulators, the BASIC PENTACYSTEINE (*BPC*) family of transcription factors has attracted considerable attention due to its critical roles in plant development and stress adaptation [14]. Extensive studies have been conducted on the polyploid evolution and stress response mechanisms in wheat and other cereals, and the *BPC* gene family has been reported in barley. However, genome-wide identification and functional characterization of the *BPC* transcription factor family remain unreported in *B. juncea*. To date, several gene families, including *MYB*, *TCP*, and *BjSWEET*, have been systematically analyzed in *B. juncea* [21,22,23], providing a solid foundation for the study of the *BPC* gene family, although its specific functions in *B. juncea* remain unclear. Therefore, dissecting the *BPC* gene family in *B. juncea* has distinct species-specificity and important theoretical and breeding value.

The model plant *Arabidopsis thaliana* harbors seven *AtBPC* genes (*AtBPC1*–*AtBPC7*), which are classified into three subfamilies based on domain architecture and sequence similarity: subfamily I (*AtBPC1*–*AtBPC3*), subfamily II (*AtBPC4*–*AtBPC6*), and subfamily III (*AtBPC7*) [24]. All AtBPC proteins share a conserved C-terminal GAGA DNA-binding domain that specifically recognizes and binds GAGA motifs in the promoters of target genes, while their N-terminal regions modulate transcriptional activation or repression [25,26]. Loss-of-function studies have shown that *AtBPC* mutations lead to dwarfism, small and curled leaves, abnormal floral organ morphology, and altered cell differentiation and senescence [24]. Functional redundancy and antagonism among family members are complex, with the *atbpc1 atbpc2* double mutant displaying only mild phenotypic defects, whereas the *atbpc1 atbpc2 atbpc4 atbpc6* quadruple mutant exhibits severe developmental abnormalities that can be rescued by *AtBPC3*. Notably, *AtBPC3* also antagonizes other *AtBPC* members in regulating circadian rhythm and flowering time [27].

Functional characterization of *BPC* homologs in other species further underscores their conserved yet diversified roles. For instance, *MdBPC2* in apple negatively regulates auxin biosynthesis to suppress vegetative growth [28]; *GhBPC4* in cotton acts as a key cold-stress response factor, and its silencing results in severe chilling injury [29]; in *Medicago truncatula*, *MtBPC1* represses *MtABI4* transcription during early seed development by binding to CT-rich motifs in the *MtABI4* promoter [30].

*BPC* gene families have been systematically identified in diverse species, including *Arabidopsis* [24], barley [31], *Brassica napus* [32,33], rice [34], cotton [29], and cucumber [35], and their expression patterns in response to abiotic stresses such as drought [32], cold [29], and phytohormones [32,35] have been partially elucidated. However, despite light quality being a pivotal environmental signal governing plant growth and development [36], the regulatory mechanisms for *BPC* gene expression in response to light spectra remain largely unexplored. In particular, the *BPC* homologs in *B. juncea* (designated *BjuBPCs*) have not been comprehensively identified or functionally characterized. A systematic bioinformatic analysis of the *BjuBPC* gene family is therefore essential to understand their roles in *B. juncea* development and environmental adaptation, and to support future molecular breeding efforts for this polyploid crop.

In this study, we performed genome-wide identification and characterization of the *BjuBPC* gene family in *B. juncea*. We analyzed gene structure, conserved domains, phylogenetic relationships, tissue-specific expression profiles, and expression responses to different light qualities and exogenous salicylic acid treatments. Our findings provide valuable insights into the functional diversification of *BPC* transcription factors in growth and stress responses and lay a foundation for uncovering the evolutionary and breeding implications of the gene family in polyploid *Brassica* species.

## 2. Results

### 2.1. Genome-Wide Identification and Physicochemical Characterization of the BPC Gene Family in B. juncea

Using the seven BPC protein sequences from *A. thaliana* as queries, a BLASTP search was performed against the *B. juncea* genome followed by validation based on the presence of the conserved GAGA DNA-binding domain (Pfam ID: PF06217). This integrated approach led to the identification of 25 *BPC* gene family members in *B. juncea*, designated *BjuBPC1* through *BjuBPC25*.

Physicochemical analysis (Table 1) revealed that the BjuBPC proteins range from 226 to 599 amino acids in length, with molecular weights between 25.09 and 68.82 kDa. Their theoretical isoelectric points (pI) vary from 8.76 to 10.12, indicating a consistently basic nature. The instability index ranges from 34.56 to 63.63; 23 members (92%) exhibit values > 40 and are thus classified as unstable proteins [37], while only BjuBPC5 and BjuBPC14 (instability index < 40) are predicted to be stable. The aliphatic index of these proteins falls between 48.57 and 71.94. Notably, all BjuBPC proteins display negative grand average of hydropathy (GRAVY) values, suggesting a hydrophilic character.

Subcellular localization prediction using Plant-mPLoc 2.0 indicated that all BjuBPC proteins are targeted to the nucleus. Combined with the presence of the conserved PF06217 domain and their nuclear localization, these findings strongly support the hypothesis that BjuBPC proteins function as transcription factors in the regulation of gene expression, consistent with the well-characterized roles of the *BPC* family in *A. thaliana* [24,27].

### 2.2. Chromosomal Localization and Synteny Analysis of the BjuBPC Gene Family

**Figure 1:**
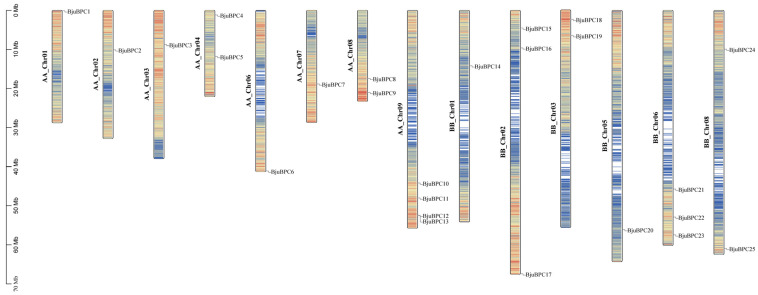
This figure shows the chromosomal locations of the 25 *BPC* gene loci in *B. juncea*. All loci are mapped to their corresponding positions across 14 of the 18 chromosomes in the *B. juncea* genome. Chromosome numbers are labeled on the left side of each chromosome, and a scale bar on the far left indicates chromosome size.

Chromosomal distribution of the *BjuBPC* genes was visualized using TBtools (Figure 1). A total of 25 *BjuBPC* genes were unevenly distributed across the 14 chromosomes of *B. juncea* and were named accordingly as *BjuBPC1*–*BjuBPC25*. Chromosome AA_Chr09 harbors the highest number of *BjuBPC* genes (four members), whereas AA_Chr01, AA_Chr02, AA_Chr03, AA_Chr06, AA_Chr07, BB_Chr01 and BB_Chr05 each contain only one gene. Two genes are located on AA_Chr04, AA_Chr08 and BB_Chr03, while three genes are found on BB_Chr02 and BB_Chr06.

**Figure 2:**
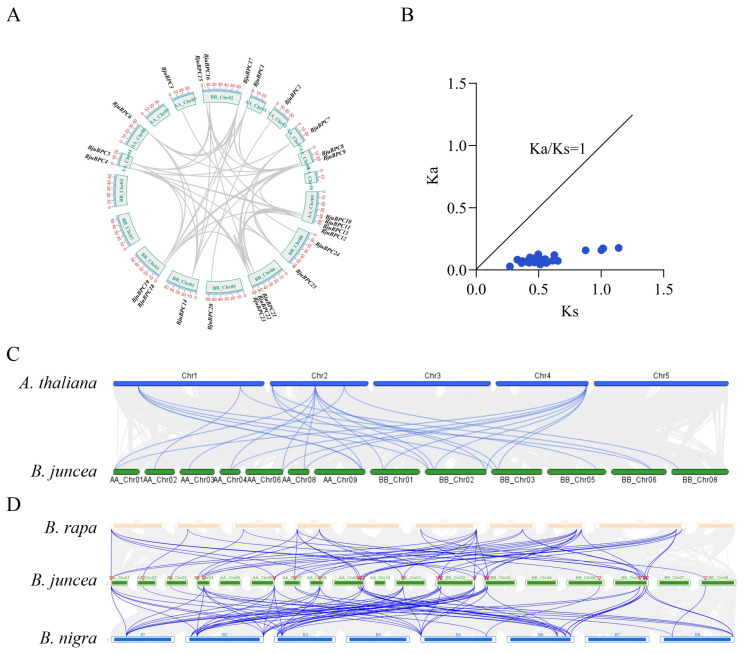
Synteny and selection pressure analysis of *BPC* genes in *B. juncea*. (**A**) Synteny analysis of *BjuBPC* genes. (**B**) Ka/Ks analysis of duplicated *BjuBPC* gene pairs. (**C**) Synteny analysis of *BPC* genes between *A. thaliana* and *B. juncea*. (**D**) Synteny analysis of *BPC* genes among *B. rapa*, *B. juncea*, and *B. nigra*.

Intra- and interspecific synteny analyses provided further insights into the evolutionary dynamics of the *BPC* gene family. Intra-genomic synteny analysis revealed that all syntenic *BjuBPC* gene pairs are located on different chromosomes (Figure 2A), with no tandem duplicates detected. This pattern indicates that the expansion of the *BjuBPC* gene family in *B. juncea* was primarily driven by whole-genome duplication (WGD) or large-scale segmental duplication events. The Ka/Ks ratios for these duplicated gene pairs range from 0.048 to 0.298 (all < 1), suggesting strong purifying selection during the evolution of this gene family (Figure 2B).

To evaluate the conservation of *BPC* genes within the Brassicaceae, interspecific synteny analyses were conducted between *B. juncea* and *A. thaliana*, *B. rapa* (A-genome donor), and *B. nigra* (B-genome donor). A total of 27 syntenic *BPC* gene pairs were identified between *B. juncea* and *A. thaliana* (Figure 2C), 53 pairs between *B. juncea* and *B. rapa*, and 39 pairs between *B. juncea* and *B. nigra* (Figure 2D). These extensive syntenic relationships underscore the high degree of functional conservation of the *BPC* gene family throughout the evolutionary history of Brassicaceae species.

### 2.3. Phylogenetic Analysis of the BjuBPC Gene Family

**Figure 3:**
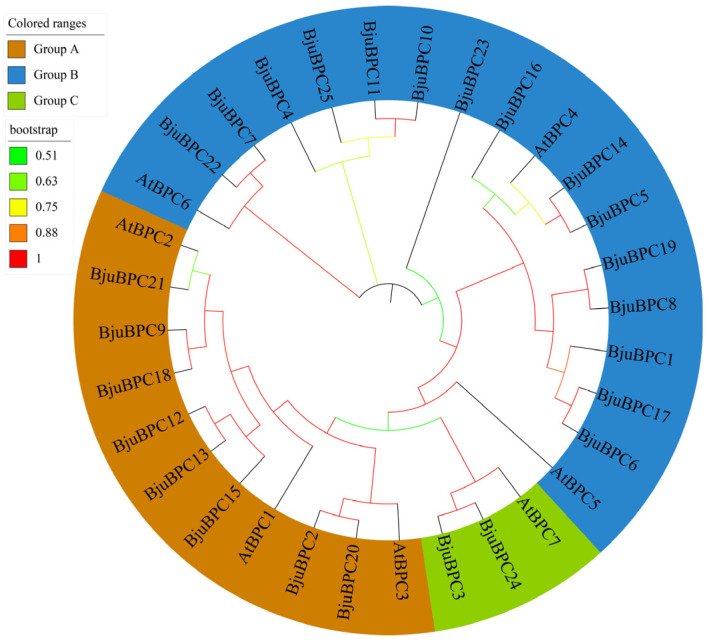
Phylogenetic tree of *BPC* gene family of *A. thaliana* and *B. juncea.* The phylogenetic tree was constructed using the BPC protein sequences from *A. thaliana* (7 genes) and *B. juncea* (25 genes) with 1000 bootstrap replicates. The genes clustered into three groups (A, B, and C). Branches are colored according to bootstrap support values, which range from 0.51 to 1.

To elucidate the evolutionary relationships within the *BjuBPC* gene family, a phylogenetic tree was constructed based on the multiple sequence alignment of BPC protein sequences from *A. thaliana* and *B. juncea* (Figure 3). The resulting topology reveals that *BjuBPC* genes cluster in a pattern highly consistent with that of their *A. thaliana* counterparts. The entire *BPC* family is divided into three distinct clades: Group A, Group B, and Group C. Specifically, Group A contained 11 members in total, including 8 *BjuBPC* genes and 3 *AtBPC* genes (*AtBPC1*-*AtBPC3*); Group B consisted of 18 members, comprising 15 *BjuBPC* genes and 3 *AtBPC* genes (*AtBPC4*-*AtBPC6*); and Group C had 3 members, which included 2 *BjuBPC* genes and 1 *AtBPC* gene (*AtBPC7*).

Notably, none of the *BjuBPC* genes grouped with *AtBPC5*, and no ortholog of *AtBPC5* was identified in the *B. juncea* genome. This suggests that the *BPC5* lineage may have been lost during the evolution of *B. juncea*, or that its homolog has diverged substantially in sequence, rendering it undetectable by current homology-based identification methods.

### 2.4. Gene Structure and Conserved-Motif Analysis of the BjuBPC Gene Family

**Figure 4:**
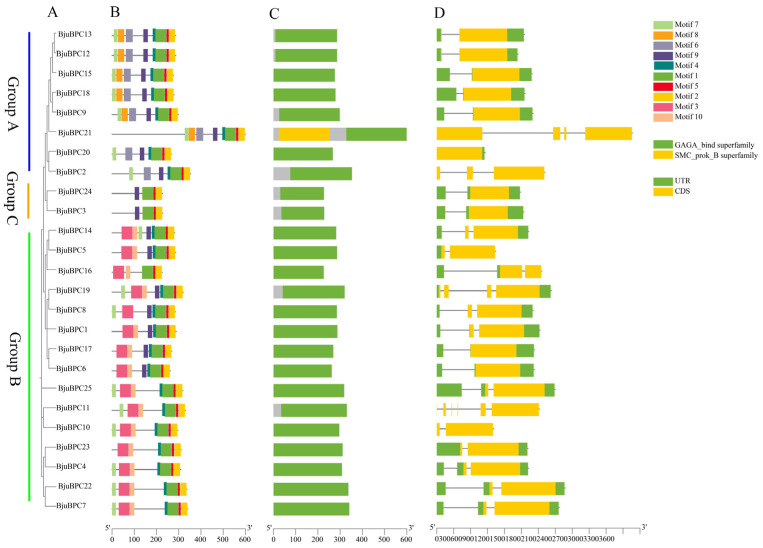
Phylogenetic analysis, conserved motifs, domains, and gene structure of *BPC* genes in *B. juncea*. (**A**) The neighbor-joining phylogenetic tree of BjuBPC proteins classifies all members into three groups (A, B, and C), denoted by differently colored lines. (**B**) Distribution of ten conserved motifs identified by MEME suite in BjuBPC proteins, with each motif uniquely colored. (**C**) Conserved protein domains, with green and yellow boxes representing the GAGA and SMC-prok domains, respectively. (**D**) Exon–intron structure of *BjuBPC* genes, where yellow and green rectangles indicate coding sequences (CDS) and untranslated regions (UTRs), respectively. The phylogenetic order of genes is consistent across all panels.

Domain architecture analysis revealed that all 25 BjuBPC proteins contain the conserved GAGA DNA-binding domain (Figure 4A,C). In addition, several members possess additional domains; for example, BjuBPC21 harbors an SMC (Structural Maintenance of Chromosomes) domain.

Gene structures within each phylogenetic subgroup are largely conserved, with only minor variations observed among a few members. To further characterize the structural features of the *BjuBPC* family, conserved motifs were identified using MEME. The top ten statistically significant motifs (Motif 1–10) were selected for detailed analysis (Figure 4B). Motif 1, Motif 2, and Motif 5 are present in all BjuBPC proteins, whereas Motif 6, Motif 7, and Motif 8 are exclusively found in Group A members, and Motif 3 and Motif 10 are exclusively found in Group B members. This subgroup-specific motif distribution strongly suggests that conserved motifs are closely linked to protein structural organization and may play critical roles in defining the functional specificity of BjuBPC proteins.

Gene structure analysis (Figure 4D) showed that 10 *BjuBPC* genes lack introns (intronless), while the remaining 15 contain 1–4 introns. Notably, all members of Group C are intronless. In Group A, all genes except *BjuBPC2* and *BjuBPC21* are intronless, whereas in Group B, all members except *BjuBPC6* and *BjuBPC17* contain introns. This pattern of structural divergence aligns closely with the phylogenetic classification, providing valuable insights into the evolutionary history of the *BjuBPC* gene family.

### 2.5. Cis-Regulatory Element Analysis of the BjuBPC Gene Family

**Figure 5:**
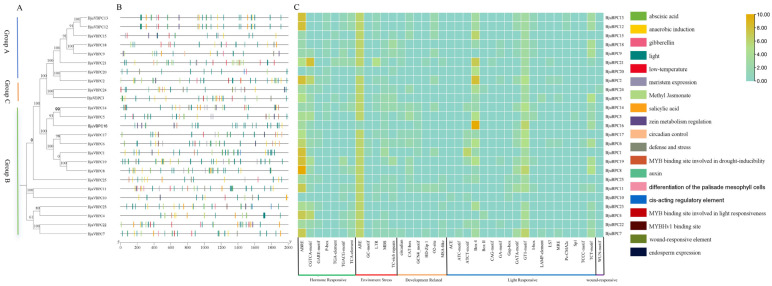
The analysis of the cis-acting element characteristics of the *BjuBPC* gene family members. (**A**) The neighbor-joining phylogenetic tree of BjuBPC proteins classifies all members into three groups (A, B, and C), denoted by differently colored lines. (**B**) The distribution and arrangement of cis-acting elements in the promoter regions, with different elements represented by colored boxes. (**C**) A heatmap displaying the number of cis-acting elements contained by members of different groups, where the bar graph shows the total content of three types of cis-acting elements, and the numbers and shades within the boxes indicate the specific quantities of each element.

To investigate the transcriptional regulatory mechanisms underlying the *BjuBPC* gene family, we analyzed the 2000 bp promoter regions upstream of the start codon for all 25 members (Figure 5). The results revealed that each *BjuBPC* promoter is enriched with diverse cis-regulatory elements, which can be broadly categorized into three functional groups: (i) abiotic-stress-responsive elements (e.g., low-temperature induction, anaerobic induction); (ii) hormone-responsive elements (e.g., ABA responsiveness, gibberellin, auxin, methyl jasmonate [MeJA], and salicylic acid [SA]); and (iii) development- and metabolism-related elements (e.g., light responsiveness, circadian control, seed storage protein regulation, cell cycle regulation, and palisade mesophyll cell differentiation).

Light-responsive elements were the most prevalent and universally present across all *BjuBPC* promoters, suggesting that the expression of this gene family is broadly modulated by light signals. ABA-responsive elements were frequently detected in multiple genes (e.g., *BjuBPC1*, *BjuBPC2*, and *BjuBPC3*), indicating their potential roles in abiotic stress responses.

Among hormone-related elements, MeJA-responsive motifs were widely distributed (e.g., in *BjuBPC1*, *BjuBPC2*, *BjuBPC3*, *BjuBPC4*, *BjuBPC21*), indicating possible involvement in defense responses. Auxin-responsive elements were identified in *BjuBPC3* and *BjuBPC21*, while SA-responsive elements were present in *BjuBPC3*, *BjuBPC4*, *BjuBPC7*, *BjuBPC9*, and *BjuBPC24*.

Notably, several *BjuBPC* promoters harbor multiple specialized regulatory motifs. For instance, *BjuBPC7* and *BjuBPC11* contain elements associated with palisade mesophyll cell differentiation, suggesting a role in leaf morphogenesis. *BjuBPC7*, *BjuBPC11*, and *BjuBPC22* possess cell cycle regulatory elements, implying functions in cell division or developmental progression. Elements annotated as “zein metabolism regulation”, originally defined in monocots, were found in *BjuBPC9*, *BjuBPC11*, *BjuBPC13*, and *BjuBPC15*, potentially reflecting conserved roles in nitrogen metabolism or seed development. Additionally, a wound-responsive element was identified in the promoter of *BjuBPC14*, hinting at its involvement in rapid responses to mechanical damage or pathogen attack. Circadian control elements were detected in *BjuBPC5*, *BjuBPC14*, and *BjuBPC17*, indicating possible regulation by the endogenous circadian clock.

Collectively, the distinct composition of cis-regulatory elements among *BjuBPC* promoters highlights substantial functional diversification within the gene family, particularly in mediating responses to environmental cues, phytohormone signaling, and developmental processes. This regulatory complexity provides critical insights into the multifaceted roles of *BPC* transcription factors in *B. juncea* growth, development, and stress adaptation.

### 2.6. Tissue-Specific Expression Profiling of the BjuBPC Gene Family

**Figure 6:**
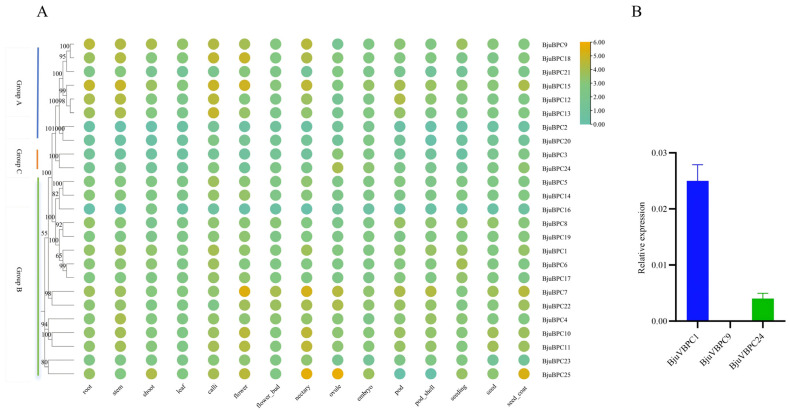
Tissue-specific expression profiles of *BPC* family genes in *B. juncea*. (**A**) Heatmap depicting the expression levels (in transcript per million, TPM) of 25 *BjuBPC* genes across 16 tissues, based on data from the BjuIR database. The heatmap was generated and row-normalized using the TBtools-II (v2.390) software, with expression levels color-coded from green (low) to brown (high). (**B**) Relative expression levels of three selected genes (*BjuBPC1*, *BjuBPC9*, and *BjuBPC24*) in leaves, as determined by qRT-PCR. The 2^−ΔΔCt^ method was used for quantification, with the average ΔCt value of these three genes serving as the calibrator.

To elucidate functional diversification within the *BjuBPC* gene family, we extracted transcript abundance data (TPM values) for all 25 members across 16 tissues (root, stem, shoot, leaf, callus, flower, floral bud, nectary, ovule, embryo, silique, silique wall, seedling, immature seed, mature seed, and seed coat) from the *B. juncea* expression matrix in the BjuIR database (https://yanglab.hzau.edu.cn/BjuIR). Hierarchical clustering of expression patterns classified the genes into four distinct groups (Figure 6A):

(1) Reproductive-stage-specific genes: *BjuBPC7*, *BjuBPC9*, *BjuBPC10*, *BjuBPC11*, *BjuBPC15*, *BjuBPC18*, and *BjuBPC25* exhibited peak expression in reproductive tissues, particularly in ovule, embryo, seed, and flower tissues. Notably, *BjuBPC25* showed the highest expression in ovule (TPM = 53.746) and embryo (TPM = 48.753) tissues, while its expression was markedly low in vegetative tissues such as root and leaf tissues. This expression pattern strongly suggests specialized roles in reproductive development, embryogenesis, and seed maturation.
(2) Vegetative growth- and basal metabolism-associated genes: *BjuBPC1*, *BjuBPC4*, *BjuBPC5*, *BjuBPC6*, *BjuBPC8*, *BjuBPC12*, *BjuBPC13*, *BjuBPC14*, *BjuBPC17*, *BjuBPC19*, *BjuBPC22*, and *BjuBPC23* displayed constitutively high expression in vegetative tissues (root, stem, shoot, leaf). For example, *BjuBPC12* and *BjuBPC13* reached peak expression in callus tissues (TPM = 21.751 and 27.184, respectively), while *BjuBPC1* and *BjuBPC4* maintained moderate-to-high expression across multiple tissues. These genes are likely involved in fundamental cellular processes, cell proliferation, and general growth maintenance.
(3) Flower organ-specific genes: *BjuBPC3*, *BjuBPC20*, *BjuBPC21*, and *BjuBPC24* were specifically enriched in floral structures, including flower, floral bud, and nectary tissues. *BjuBPC24* showed the highest expression in ovule tissues (TPM = 15.109), with notable signals also noted in floral bud (TPM = 4.077) and nectary (TPM = 3.075) tissues. Although ovule expression was prominent, its overall expression profile aligns more closely with floral organ identity, suggesting potential roles in flower development, pollination, or floral-specific secondary metabolism.
(4) Low-expression or conditionally activated genes: *BjuBPC2*, *BjuBPC16*, and to some extent *BjuBPC20* formed a cluster characterized by extremely low expression across all sampled tissues (mostly < 1.0 TPM). *BjuBPC2* remained below 1.0 TPM in every tissue, while *BjuBPC16* showed minimal expression except for a modest increase in shoot tissues (TPM = 3.667). These genes may represent pseudogenes, functionally redundant paralogs, or loci under stringent epigenetic repression, potentially activated only under specific environmental or developmental conditions not captured in this dataset.

To validate the RNA-seq data, qRT-PCR was performed on leaf samples for selected genes. As shown in Figure 6B, the expression levels of *BjuBPC1*, *BjuBPC9*, and *BjuBPC24* measured by qRT-PCR were consistent with their transcriptomic profiles, confirming the reliability of the BjuIR expression dataset.

### 2.7. Expression Analysis of BjuBPC Genes Under Different Light Qualities and Salicylic Acid Treatment

**Figure 7:**
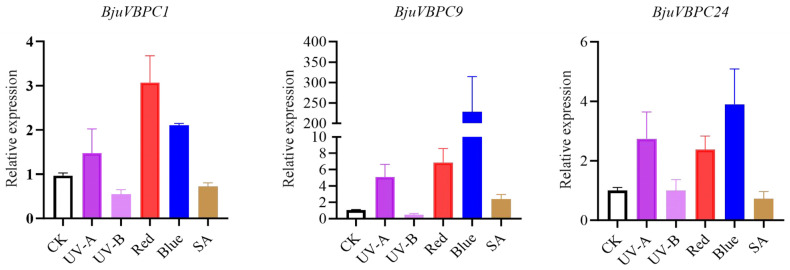
Expression of *BjuBPC* gene in mustard induced by salicylic acid and different light qualities. The control (CK) included leaves from the 6-leaf–1-heart stage under white light, with its gene expression level normalized to 1. Experimental groups were induced under UV-A, UV-B, red, or blue light. The 2^−ΔΔCt^ method was used for quantification. All data represent three biological replicates.

*BjuBPC1*, *BjuBPC9*, and *BjuBPC24* belong to subgroups B, A, and C, respectively. All three promoters contain light-responsive cis-elements, whereas salicylic acid (SA)-responsive elements are present only in the promoters of *BjuBPC9* and *BjuBPC24*, but absent in that of *BjuBPC1*. To test whether these cis-elements functionally mediate responses to light and SA, we examined the relative expression levels of these three genes in *B. juncea* seedlings subjected to four light treatments (UV-A, UV-B, red light, and blue light) and exogenous SA application. Expression levels were normalized to those of the control (CK) (Figure 7).

*BjuBPC1*: Expression was significantly upregulated under UV-A, red light, and blue light, reaching 1.47-, 3.07-, and 2.10 times that of the control, respectively. In contrast, UV-B treatment strongly suppressed its expression to 0.54 times that of the control. SA treatment had no significant effect, with expression reduced only slightly to 0.72 times that of the control, consistent with the absence of SA-responsive elements in its promoter.

*BjuBPC9*: This gene exhibited the most pronounced response among the three. Its expression was markedly induced by UV-A (5.11-fold), red light (6.88-fold), blue light (228.26-fold), and SA (2.41-fold) compared to the control. The exceptionally high induction under blue light suggests a highly specific blue light-mediated regulatory mechanism. Conversely, UV-B treatment significantly repressed *BjuBPC9* expression to 0.48 times that of the control.

*BjuBPC24*: Expression was significantly enhanced under UV-A (2.73-fold), red light (3.32-fold), and blue light (3.90-fold). However, SA treatment did not significantly alter its expression (0.72-fold relative to the control), despite the presence of an SA-responsive element in its promoter. UV-B treatment showed no significant effect on *BjuBPC24* expression.

These results demonstrate that the presence of light-responsive cis-elements correlates well with transcriptional activation under specific light conditions, while the functional relevance of SA-responsive elements may be context-dependent or require additional co-regulators for full activation.

## 3. Discussion

Plant traits are shaped by the interplay between genetic programs and environmental cues. Upon perceiving external signals, plants activate transcription-factor-centered regulatory networks to precisely coordinate gene expression and achieve environmental adaptation. *BPC* transcription factors play pivotal roles in this process, particularly in modulating developmental plasticity and tolerance to abiotic stresses. To date, the *BPC* gene family has been systematically identified and functionally characterized in multiple species, including Arabidopsis [24], barley [31], *Brassica napus* [32,33], rice [34], cotton [29], and cucumber [35]. Despite the agricultural and medicinal importance of *B. juncea*, and growing interest in its growth, development, and stress response mechanisms [38,39,40,41,42,43], the *BjuBPC* gene family has not yet been comprehensively investigated at the genome-wide level. In this study, we identified 25 *BjuBPC* genes in *B. juncea* and conducted an integrated analysis of their physicochemical properties, gene structures, phylogenetic relationships, and expression patterns in response to distinct light qualities (UV-A, UV-B, blue light, red light) and exogenous salicylic acid (SA). Our work not only fills a critical gap in knowledge of the functional genomics of *B. juncea* but also provides a foundational framework for dissecting the molecular mechanisms by which *BPC* transcription factors regulate development and environmental adaptation in this polyploid crop.

*B. juncea* is an allotetraploid (AABB, 2*n* = 36) that originated from natural hybridization and whole-genome duplication between the diploid progenitors *B. rapa* (A-genome) and *B. nigra* (B-genome) [6]. Population structure, principal component, and phylogenetic analyses consistently classify *B. juncea* into three major types comprising six genetic groups, reflecting its exceptionally high genetic diversity [6]. Genomic studies further indicate that the A-subgenome (derived from *B. rapa*) exhibits greater genetic variation than the B-subgenome (from *B. nigra*) [6,9,44]. Consistent with this evolutionary context, we identified 11 and 13 *BPC* genes in the A- and B-genomes of the diploid progenitors, respectively, and 13 and 12 *BjuBPC* genes in the corresponding A- and B-subgenomes of *B. juncea*. The relatively balanced retention of *BPC* family members across subgenomes suggests limited gene loss or gain following polyploidization. Moreover, synteny analysis revealed 53 collinear gene pairs between *B. rapa* (A-genome) and *B. juncea*, and 39 such pairs between *B. nigra* (B-genome) and *B. juncea* (Figure 2D), underscoring the high degree of conservation of the *BPC* family throughout *Brassica* evolution. The phylogenetic clustering pattern of *BjuBPC* genes closely mirrors that of *AtBPC* genes (Figure 4), further supporting an ancient origin and structural conservation of this family within the Brassicaceae. Notably, no ortholog of *AtBPC5*, a pseudogene in *A. thaliana* [24,25], was detected in the *B. juncea* genome. This absence suggests that the *BPC5* lineage may have been selectively lost during the evolutionary trajectory of *B. juncea*. A similar loss of specific *BPC* paralogs has been reported in *B. napus* [33], indicating that certain *BPC* members may be dispensable or functionally redundant in polyploid *Brassica* species, leading to their elimination under purifying selection or genomic rearrangement.

Promoters function as molecular switches that govern transcriptional initiation and mediate gene responses to developmental cues and environmental signals [45,46,47,48]. In this study, comprehensive in silico analysis revealed abundant cis-regulatory elements associated with light, phytohormones, and stress responses within the promoters of the *BjuBPC* gene family. Notably, light-responsive elements were universally present across all *BjuBPC* promoters, suggesting that light signaling constitutes a fundamental regulatory layer for the entire gene family. Members exhibiting high expression in reproductive tissues, such as *BjuBPC7*, *BjuBPC9*, and *BjuBPC25*, harbored enriched seed development- and cell-cycle-related motifs in their promoters, consistent with the established roles of Arabidopsis *AtBPC1/2* in reproductive development. By contrast, genes displaying constitutive expression in vegetative organs (e.g., *BjuBPC12* and *BjuBPC13*) predominantly contain light-responsive and basal metabolic elements, implying their involvement in sustaining fundamental cellular processes during vegetative growth. Several flower-preferential genes, exemplified by *BjuBPC24*, possessed promoters enriched with methyl jasmonate- and auxin-responsive elements, potentially linking their transcriptional activity to floral development or pollination-related processes. Intriguingly, certain members, including *BjuBPC2*, *BjuBPC16*, and *BjuBPC20*, showed minimal expression across all examined tissues despite harboring diverse stress- and hormone-responsive elements, indicating that their transcription may be under stringent repression and activated only by specific developmental or environmental triggers. *BjuBPC14* represents a distinct case: its promoter contains wound-responsive and circadian-rhythm-related elements, yet the gene displays constitutive expression in vegetative tissues. This configuration may enable coordinated regulation between basal defense responses and the circadian clock, although functional validation remains necessary to confirm this hypothesis.

Light serves as a pivotal environmental cue that orchestrates plant growth and development, primary metabolism, and the accumulation of secondary metabolites [49]. Natural sunlight comprises a composite spectrum of visible-light, ultraviolet (UV), and infrared radiation. Within this spectrum, visible wavelengths, particularly red and blue light, drive photosynthesis and mediate photomorphogenic signaling [49], whereas UV radiation (UV-A and UV-B) plays a distinctive role in modulating secondary metabolism [49,50,51,52]. Beyond light signals, phytohormones critically regulate developmental transitions and metabolic reprogramming [53]; salicylic acid (SA), for instance, function as a central mediator of abiotic stress responses and associated physiological adjustments [54,55]. In silico promoter analysis of *BjuBPC* genes in *B. juncea* revealed a ubiquitous presence of light-responsive cis-elements, with SA-responsive elements (TCA-elements) distributed in a gene-specific manner. Notably, the promoter of *BjuBPC1* lacks a canonical TCA-element, whereas *BjuBPC9* and *BjuBPC24* possess such elements. To dissect the functional implications of this structural variation, we selected these three representative genes, spanning distinct phylogenetic subgroups, for expression profiling under exogenous SA treatment and four monochromatic light conditions (UV-A, UV-B, red, and blue light). Upon SA application, *BjuBPC1*, *BjuBPC9*, and *BjuBPC24* exhibited modest transcriptional changes relative to their pronounced responses under light treatments (Figure 7), suggesting that the *BjuBPC* gene family primarily functions in developmental regulation rather than in orchestrating defense-associated secondary metabolism. All three genes displayed a consistent response pattern to UV radiation: UV-A exposure upregulated their expression, whereas UV-B suppressed transcription. This dichotomy aligns with established physiological roles of UV wavelengths, whereby UV-A generally promotes growth and photomorphogenesis, while UV-B triggers defense metabolism [50,51,52,56], further supporting the involvement of *BjuBPC* genes in sustaining basal growth processes. Both red and blue light markedly induced the expression of all three genes. Particularly striking was the 228.26-fold upregulation of *BjuBPC9* under blue light relative to the control, a response magnitude substantially exceeding that elicited by other wavelengths. This exceptional sensitivity positions *BjuBPC9* as a potential hub in blue-light signaling, possibly acting downstream of cryptochrome photoreceptors (e.g., CRY1) to mediate large-scale transcriptional reprogramming. Collectively, the differential responsiveness of *BjuBPC* genes to SA versus specific light qualities reflects a sophisticated regulatory strategy whereby plants prioritize growth-promoting light signals under non-stress conditions while retaining the capacity to modulate gene expression in response to stress-associated cues such as SA. This balance enables optimal resource allocation between development and defense.

In summary, the *BjuBPC* gene family in *B. juncea* has evolved beyond its canonical role in developmental regulation to function as a potential integrator of environmental cues (light and hormone signals) and developmental programs. This study provides insights into the molecular mechanisms underlying environmental adaptation in polyploid crops and establishes a foundation for future functional characterization of *BjuBPC* genes in *B. juncea*.

## 4. Material and Methods

### 4.1. Plant Materials, Light, and Hormone Treatments

A self-incompatible inbred line of root mustard (*Brassica juncea*) was used as the experimental material. Seeds were sown and seedlings were grown in a controlled-environment growth chamber under a 16 h light/8 h dark photoperiod at 22 °C. At the six-leaf–one-heart stage, seedlings were subjected to the following treatments, all maintained at 22 °C: (i) white light (control, CK), (ii) ultraviolet-A (UV-A), (iii) ultraviolet-B (UV-B), (iv) red light (Red), and (v) blue light (Blue).

Each light treatment was applied for 16 h per day over a period of 7 days. Light sources and spectral ranges were as follows: white light (full spectrum, 400–700 nm); UV-A (340 nm); UV-B (313 nm); red light (660 nm); blue light (450 nm). The photosynthetic photon flux density (PPFD) at the plant canopy was 225 μmol·m^−2^·s^−1^, with a distance of 20 cm from the light source to the leaves, and this condition was applied to all treatments. In parallel, a separate group of seedlings was treated with 0.5 μmol/L [57] salicylic acid (SA) (Shanghai Yuanye Biotechnology Co., Ltd., Shanghai, China; Cat. No. A10050) solution for 6 h. During the treatment, the salicylic acid solution was evenly sprayed onto the adaxial surface of *B. juncea* leaves, until the leaves were uniformly moistened without runoff. At the end of each treatment, leaf tissues were immediately harvested, flash-frozen in liquid nitrogen, and stored at −80 °C until further analysis.

### 4.2. Data Sources

Genome sequences of *B. juncea*, *B. rapa*, and *B. nigra*, as well as *B. juncea* transcriptome data, were retrieved from the BjuIR database (https://yanglab.hzau.edu.cn/BjuIR, accessed on 12 January 2026; see Supplementary Table S1 [9,58,59]). The protein sequences of *Arabidopsis thaliana BPC* family members were downloaded from The Arabidopsis Information Resource (TAIR; https://www.arabidopsis.org, accessed on 8 October 2025).

### 4.3. Identification and Physicochemical Characterization of the BPC Gene Family in Brassica juncea

The seven BPC protein sequences from *A. thaliana* were used as queries to perform BLASTP searches against the *B. juncea* T84-66 V2.0 proteome database (BjuIR, https://yanglab.hzau.edu.cn/BjuIR/blast, accessed on 8 October 2025) with an E-value cutoff of 1 × 10^−20^. Redundant hits were removed, yielding an initial set of candidate BPC homologs in *B. juncea*. To complement this homology-based approach, a hidden Markov model (HMM) profile for the conserved GAGA DNA-binding domain (Pfam ID: PF06217) was retrieved from the Pfam database (http://pfam.xfam.org/, accessed on 8 October 2025) [60] and used to scan the entire *B. juncea* proteome via the HMMER web server (https://www.ebi.ac.uk/Tools/hmmer/search/hmmscan, accessed on 8 October 2025).

All candidate proteins containing the GAGA domain were further validated using the NCBI Batch CD-Search tool (https://www.ncbi.nlm.nih.gov/Structure/bwrpsb/bwrpsb.cgi, accessed on 8 October 2025) to assess domain integrity. Sequences with truncated or incomplete GAGA domains were manually excluded. A total of 25 non-redundant *B. juncea BPC* genes were finally identified and designated *BjuBPC1* through *BjuBPC25*. Their corresponding coding sequences (CDS) and genomic sequences were extracted for downstream analyses.

Physicochemical properties of the BjuBPC proteins, including amino acid length, molecular weight (kDa), theoretical isoelectric point (pI), instability index, and grand average of hydropathicity (GRAVY), were predicted using the ProtParam tool on the ExPASy platform (http://web.expasy.org/protparam/, accessed on 8 October 2025). Subcellular localization was predicted using Plant-mPLoc 2.0 (http://www.csbio.sjtu.edu.cn/bioinf/plant-multi/, accessed on 8 October 2025).

### 4.4. Chromosomal Localization and Synteny Analysis of BjuBPC Genes

Chromosomal positions of the *BjuBPC* genes were mapped and visualized using TBtools-II (v2.390) [61]. Intra-genomic synteny within *B. juncea* was analyzed using the MCScanX module integrated in TBtools-II to identify duplication events. Interspecific synteny relationships were further investigated between *B. juncea* and three reference genomes: *A. thaliana*, *B. rapa*, and *B. nigra*. For each pair of duplicated *BjuBPC* genes, the non-synonymous substitution rate (Ka), synonymous substitution rate (Ks), and Ka/Ks ratio were calculated using the KaKs_Calculator plugin in TBtools-II to infer the type and strength of selective pressure during evolution.

### 4.5. Phylogenetic Analysis of the BPC Gene Family

Multiple sequence alignments of BPC protein sequences from *A. thaliana* and *B. juncea* were performed using the MUSCLE algorithm implemented in MEGA12 [62]. A neighbor-joining (NJ) phylogenetic tree was constructed under the Poisson model with pairwise deletion of gaps, and branch support was assessed by 1000 bootstrap replicates. This interspecific tree was used to infer orthologous and paralogous relationships among *BPC* genes [32]. The tree was visualized and annotated using the Interactive Tree Of Life (iTOL) platform (https://itol.embl.de, accessed on 25 January 2026).

Additionally, an intra-species NJ phylogenetic tree was generated using only the *B. juncea* BPC protein sequences, applying identical parameters (Poisson model, pairwise deletion, 1000 bootstrap replicates). This tree served as the basis for classifying the *BjuBPC* gene family into distinct subgroups.

### 4.6. Gene Structure and Conserved-Motif Analysis

Conserved motifs in BjuBPC proteins were identified using the MEME Suite plugin in TBtools-II, with the following settings: maximum number of motifs = 10, motif width = 6–50 amino acids. Motifs commonly present across most family members were prioritized for functional interpretation.

Promoter regions (2000 bp upstream of the transcription start site) of all *BjuBPC* genes were extracted and submitted to the PlantCARE database (http://bioinformatics.psb.ugent.be/webtools/plantcare/html/, accessed on 8 October 2025) for prediction of cis-regulatory elements. Elements annotated as “unknown” or associated only with basal transcription (e.g., TATA-box, CAAT-box) were excluded. Only cis-elements related to hormone responsiveness, stress responses, or tissue-specific expression were retained for further analysis.

Finally, an integrated diagram depicting phylogenetic relationships, conserved-motif composition, and exon–intron structures of the *BjuBPC* genes was generated using the visualization module in TBtools-II to explore potential links between gene structure and functional divergence.

### 4.7. Expression Profiling of BjuBPC Genes Across Tissues

Transcript per million (TPM) values for *BjuBPC* genes in various tissues were downloaded from the BjuIR database (https://yanglab.hzau.edu.cn/BjuIR). A hierarchical clustering heatmap was generated using the Heatmap module in TBtools-II (v2.390) to visualize tissue-specific expression patterns.

### 4.8. qRT-PCR Analysis of BjuBPC Gene Expression Under Different Light Qualities and Salicylic Acid Treatment

Total RNA was extracted from leaf tissues collected after the treatments described in the section “Plant materials, light, and hormone treatments” using the Maxwell^®^ RSC simplyRNA Tissue Kit (Promega, Madison, WI, USA; Cat. No. AS1340). First-strand cDNA was synthesized from 1 μg of total RNA using the PrimeScript™ RT reagent Kit (Perfect Real Time; Takara, Kyoto, Japan; Cat. No. RR037A) according to the manufacturer’s instructions.

Quantitative real-time PCR (qRT-PCR) was performed on a Bio-Rad CFX real-time PCR system (Bio-Rad, Hercules, CA, USA) using TB Green™ Premix Ex Taq™ II (Takara, Kyoto, Japan; Cat. No. RR820A) with a 1:50 dilution of cDNA as a template. *B. juncea GAPDH* [63] was used as an internal reference gene. Each sample included three biological replicates, and the primer sequences are listed in Supplementary Table S2. Relative expression levels of *BjuBPC* genes were calculated using the 2^−ΔΔCt^ method. Data processing was carried out in Microsoft Excel 2019 (Microsoft, Redmond, WA, USA), and graphs were generated using GraphPad Prism 10 (GraphPad Software, San Diego, CA, USA).

## Figures and Tables

**Table 1:** The protein physicochemical properties of the BPC gene family in *B. juncea*.

Locus IDs	Gene Name	Amino Acid Number	PI	Molecular Weight/kDa	Instability Index	Aliphatic Index	GRAVY	Subcellular Localization	Clade	Subgenome
*BjuVA02G19740*	*BjuBPC2*	353	9.84	39.86	53.33	66.01	−0.635	Nucleus	A	A
*BjuVA08G32310*	*BjuBPC9*	298	9.56	33.42	53.26	54.30	−0.785	Nucleus	A	A
*BjuVA09G61830*	*BjuBPC12*	286	9.60	31.96	51.99	52.20	−0.749	Nucleus	A	A
*BjuVA09G61970*	*BjuBPC13*	286	9.60	31.96	51.99	52.20	−0.749	Nucleus	A	A
*BjuVB02G07240*	*BjuBPC15*	276	9.65	31.02	50.65	51.27	−0.856	Nucleus	A	B
*BjuVB03G05880*	*BjuBPC18*	279	9.38	31.26	54.39	48.57	−0.918	Nucleus	A	B
*BjuVB05G51790*	*BjuBPC20*	267	9.52	29.96	51.65	60.97	−0.642	Nucleus	A	B
*BjuVB06G33710*	*BjuBPC21*	599	8.76	68.82	53.96	71.94	−0.747	Nucleus	A	B
*BjuVA01G00210*	*BjuBPC1*	288	9.37	32.42	53.88	62.74	−0.899	Nucleus	B	A
*BjuVA04G01920*	*BjuBPC4*	308	9.35	34.53	57.85	57.44	−0.93	Nucleus	B	A
*BjuVA04G16390*	*BjuBPC5*	286	9.08	31.89	34.56	70.70	−0.688	Nucleus	B	A
*BjuVA06G48080*	*BjuBPC6*	262	9.27	29.68	57.63	66.37	−0.833	Nucleus	B	A
*BjuVA07G25180*	*BjuBPC7*	341	9.28	38.03	52.71	57.01	−0.859	Nucleus	B	A
*BjuVA08G24480*	*BjuBPC8*	285	9.51	31.97	54.92	54.92	−0.922	Nucleus	B	A
*BjuVA09G44830*	*BjuBPC10*	296	9.50	33.29	52.56	54.49	−0.969	Nucleus	B	A
*BjuVA09G52410*	*BjuBPC11*	330	9.50	37.14	52.76	58.33	−0.886	Nucleus	B	A
*BjuVB01G19350*	*BjuBPC14*	282	9.10	31.41	38.58	65.78	−0.743	Nucleus	B	B
*BjuVB02G12980*	*BjuBPC16*	226	10.12	25.10	47.75	67.04	−0.738	Nucleus	B	B
*BjuVB02G75810*	*BjuBPC17*	269	9.35	30.41	63.63	63.90	−0.849	Nucleus	B	B
*BjuVB03G14980*	*BjuBPC19*	320	9.64	35.76	51.34	70.78	−0.699	Nucleus	B	B
*BjuVB06G45780*	*BjuBPC22*	337	9.13	37.86	50.91	54.78	−0.943	Nucleus	B	B
*BjuVB06G53830*	*BjuBPC23*	311	8.83	35.13	55.66	57.49	−0.922	Nucleus	B	B
*BjuVB08G57020*	*BjuBPC25*	318	9.40	35.86	56.14	53.18	−1.009	Nucleus	B	B
*BjuVA03G19180*	*BjuBPC3*	228	9.82	25.17	55.04	66.67	−0.488	Nucleus	C	A
*BjuVB08G18130*	*BjuBPC24*	227	9.87	25.09	60.78	66.08	−0.523	Nucleus	C	B

## Data Availability

All data generated or analyzed during this study are included in this published article and its Supplementary Materials.

## Supplementary Materials

The following supporting information can be downloaded at: https://www.mdpi.com/article/10.3390/ijms27062664/s1.
