# Supplementary material for: Genome-Wide Identification of the BPC Gene Family in Brassica juncea and Expression Analysis of Its Regulatory Mechanisms in Response to Light and Salicylic Acid
Source: Int J Mol Sci. 2026 Mar 14;27(6):2664. doi: 10.3390/ijms27062664 (PMC13026133; doi:10.3390/ijms27062664)
Supplement: Supplementary file 1 [file ijms-27-02664-s001.zip › Table S1.pdf]

Table S1. Reference genome information

Abbr	Species	Version	Source	Database
Bra_Chiifu.v4.0	*Brassica rapa*	V4.0	[58]	BjuIR
Ni100 LR v2.0	*Brassica nigra*	v2.0	[59]	BjuIR
T84-66.V2.0	*Brassica juncea*	V2.0	[9]	BjuIR
