# Supplementary material for: Genome-Wide Identification of the BPC Gene Family in Brassica juncea and Expression Analysis of Its Regulatory Mechanisms in Response to Light and Salicylic Acid
Source: Int J Mol Sci. 2026 Mar 14;27(6):2664. doi: 10.3390/ijms27062664 (PMC13026133; doi:10.3390/ijms27062664)
Supplement: Supplementary file 1 [file ijms-27-02664-s001.zip › Table S2.pdf]

Table S2. Primers for qRT-PCR analysis

Gene name	Forward primer sequence（5′-3′）	Reverse primer sequence（5′-3′）
BjuGAPDH	GGTGCCAAGAAGGTTGTCAT	TGAGTAGCAGTGATAGAGTGGACG
BjuBPC1	GGACAGTAACGATGTCGGCT	TCTGGGGAAGCGGATACTGA
BjuBPC9	AACGGGTCATACCACCAACC	CGCACTTAACCGGGTTTTCC
BjuBPC24	GGCTTGCCTACCTCTAATGCT	GCTTCGTGGACTTCTGCTCA
